# Experimental and Computational Investigation on the Interaction of Anticancer Drug Gemcitabine with Human Plasma Protein: Effect of Copresence of Ibuprofen on the Binding

**DOI:** 10.3390/molecules27051635

**Published:** 2022-03-01

**Authors:** Mohd Sajid Ali, Hamad A. Al-Lohedan

**Affiliations:** Department of Chemistry, College of Science, King Saud University, P.O. Box 2455, Riyadh 11451, Saudi Arabia; hlohedan@ksu.edu.sa

**Keywords:** gemcitabine, serum albumin, ibuprofen, molecular docking, competitive binding, fluorescence quenching

## Abstract

The interaction of common anticancer drug gemcitabine with human serum albumin (HSA) has been studied in detail. The effect of an omnipresent nonsteroidal anti-inflammatory drug ibuprofen was also seen on the binding of HSA and gemcitabine. A slight hyperchromic shift in the difference UV-visible absorption spectra of HSA on the addition of gemcitabine gave a primary idea of the possible complex formation between them. The inner filter effect, which happens due to the significant absorbance of the ligand at the excitation and/or emission wavelengths, played an important role in the observed fluorescence quenching of HSA by gemcitabine that can be understood by comparing the observed and corrected fluorescence intensities obtained at λ_ex_ = 280 nm and 295 nm. Gemcitabine showed weak interaction with HSA, which took place via a dynamic quenching mechanism with 1:1 cooperative binding between them. Secondary structural analysis, based on circular dichroism (CD) spectroscopy, showed that low concentrations of gemcitabine did not affect the native structure of protein; however, higher concentrations affected it slightly with partial unfolding. For understanding the binding site of gemcitabine within HSA, both experimental (using site markers, warfarin and ibuprofen) as well as computational methods were employed, which revealed that the gemcitabine binding site is located between the interface of subdomain IIA and IIB within the close proximity of the warfarin site (drug site 1). The effect of ibuprofen on the binding was further elaborated because of the possibility of its coexistence with gemcitabine in the prescription given to the cancer patients, and it was noticed that, ibuprofen, even present in high amounts, did not affect the binding efficacy of gemcitabine with HSA. DFT analyses of various conformers of gemcitabine obtained from its docking with various structures of HSA (free and bounded with site markers), show that the stability of the gemcitabine molecule increased slightly after binding with ibuprofen-complexed HSA. Both experimental as well as computational results were in good agreement with each other.

## 1. Introduction

Gemcitabine is one of the most important and most prescribed medicines used to treat various types of cancers, for instance, breast cancer, ovarian cancer, non-small cell lung cancer, pancreatic cancer and bladder cancer. Chemically, it is a nucleoside analogue of deoxycytidine having two additional fluorine atoms (2′,2′-difluorodeoxycytidine) instead of hydrogen atoms in the former. Gemcitabine irreversibly inhibits ribonucleotide reductase and induces S phase arrest that leads to apoptosis [1]. It is very effective in pancreatic cancer and administrated alone or in combination of several other antineoplastic agents, such as capecitabine, paclitaxel and tyrosine kinase inhibitors [2,3,4,5]. The combination of gemcitabine along with cisplatin is very effective as compared to the combination of other cytotoxic agents with the latter in non-small lung cancer and bladder cancer [6,7]; further, its combination with other drugs produced better results in the treatment of advanced or metastatic breast cancer [8].

Apart from promising cytotoxic effects, gemcitabine is also associated with several side effects including cutaneous toxicities, pulmonary toxicity, fever, edema anemia, neutropenia, and thrombocytopenia, etc. [9]. The mode of administration of gemcitabine is principally intravenous; although, its hepatic artery infusion has also been reported in the case of tumors confined to the liver [10], which reduces its plasma toxicity.

The encounters of the drugs, particularly those that have been given intravenously or intramuscularly to the plasma protein, are obvious. The plasma proteins that commonly binds to the various endogenous or exogenous substances are serum albumin; lipoprotein; glycoprotein; and α, β‚ and γ globulins. Serum albumin is among the most abundant protein constituent of the blood (50–60%) and is an important factor in the regulation of plasma volume and tissue fluid balance. Serum albumin is also the principal carrier proteins of the blood that circulates drugs and other substances throughout the body. Due to a large number of applications, serum albumins are among the most extensively studied proteins over several past decades [11,12,13,14,15,16,17,18,19,20]. The interactions of serum albumin with drugs play an important role in determining the pharmacokinetic and pharmacodynamic actions of the latter. Stronger interactions lead to a longer stay of the drug inside the body and vice versa. The longer stay of a drug may give rise to more side effects, whereas a shorter stay might restrict the desired therapeutic effects. Moreover, drugs or any other ligand, when interacting with albumins, show the diverse range of effects on the latter; for instance, they (i) may lead to denaturation or unfolding, (ii) might trigger some partial impact on the structure and stability, (iii) did not affect their structure at all or (iv) can increase the stability of the former, depending on the structural and binding properties of the former [21,22,23,24,25,26,27,28,29,30,31,32]. Thus, it is important to know the interaction of a drug with serum albumin. As we have discussed that gemcitabine is, primarily, given through injection or infusion into the veins, there are likelihoods of its interaction with plasma proteins. Furthermore, there are opportunities to use painkiller medicines along, with the chemotherapeutic agent, to reduce the pain due to the cancer. Thus, it would also be interesting to see the effect of such drug, which is simultaneously present with another drug in the plasma and which could influence its binding with serum albumin. Ibuprofen is a common non-steroidal anti-inflammatory drug and is given for pain, fever, and inflammation. It is effective in treating painful menstrual periods, migraines and rheumatoid arthritis. It is listed in the WHO’s list of essential medicines, and, in 2019, it was among top 30 prescribed medicines in US. The coexistence of ibuprofen with the gemcitabine in cancer treatment is reported in some studies [33,34,35]. Hence, understanding the mechanism of the binding of gemcitabine with human serum albumin (HSA) in the presence of ibuprofen will also be interesting. 

Recently, we have studied the interaction of gemcitabine with model protein bovine serum albumin and lysozyme [36,37], and this work has been designed to see the interaction of gemcitabine with HSA and also to see the effect of ibuprofen on the binding. Although, Kandagal and coworkers [38] have studied the interaction of the HSA with gemcitabine using several spectroscopic techniques, several important considerations were left or unnoticed in their study, for example, the correction of the inner filter effect, which is necessary before the analysis of fluorescence data provided that the ligand has considerable absorption at the excitation and emission wavelengths [21,22,23,24,39]. The inner filter effect is a phenomenon that is usually observed in fluorescence spectroscopy and has a strong effect, particularly when the sample has the strong absorption at the excitation and/or emission wavelengths that may attenuate these and consequently affect the actual results. Further, a difference UV-visible spectrum gives more straightforward information about the ligand-induced changes in the protein rather than the simple one [21]. In addition, molecular docking is an important method in computer-aided drug design due to its uncomplicated operations and commands that allows users to get results instantly as compared to other computational methods [40]. Although, in a recent study, the binding of gemcitabine with HSA was seen using 10 ns molecular dynamics simulation [41], but this study was designed to see the stability of the drug-HSA complex when the drug was bounded to site 1 and site 2. In the present study, we have seen the detailed binding of HSA with gemcitabine along with the effect of ibuprofen on the binding using experimental, as well as computational, methods. 

## 2. Results and Discussions

### 2.1. UV-Visible Absorption Studies

**Figure 1:**
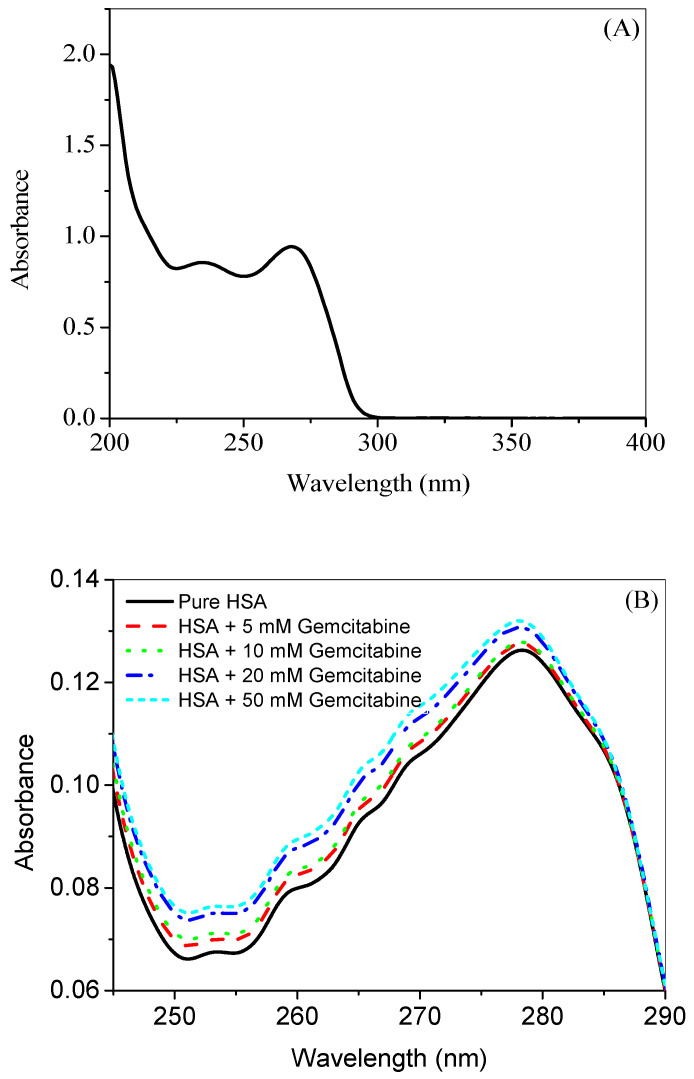
(**A**) UV-visible spectrum of 100 µM gemcitabine in 20 mM tris buffer of pH 7.4 at 25 °C. (**B**) Difference UV-visible spectra (in the range of 245 nm to 290 nm) of HSA (3 µM) in the absence and presence of several concentrations of gemcitabine in 20 mM tris buffer of pH 7.4 at 25 °C.

The UV absorption spectrum of pure gemcitabine in 20 mM tris buffer of pH 7.4 is given in Figure 1A, which shows that gemcitabine has prominent absorption between 200 nm to 280 nm, followed by a sharp decline until it becomes negligible at around 300 nm. The effect of gemcitabine on the UV-visible absorption profile of HSA is displayed in Figure 1B. When a small molecule or ligand interacts with biomolecules, the changes in the UV-visible absorption profiles of the latter are expected due to its complex formation with the former [42]. It can be seen from Figure 1B that, with the successive addition of gemcitabine, a hyperchromic shift is occurring in the difference UV-visible spectra of HSA, which is attributed to the complex formation between them [32]. 

### 2.2. Fluorescence Quenching of HSA by Gemcitabine

**Figure 2:**
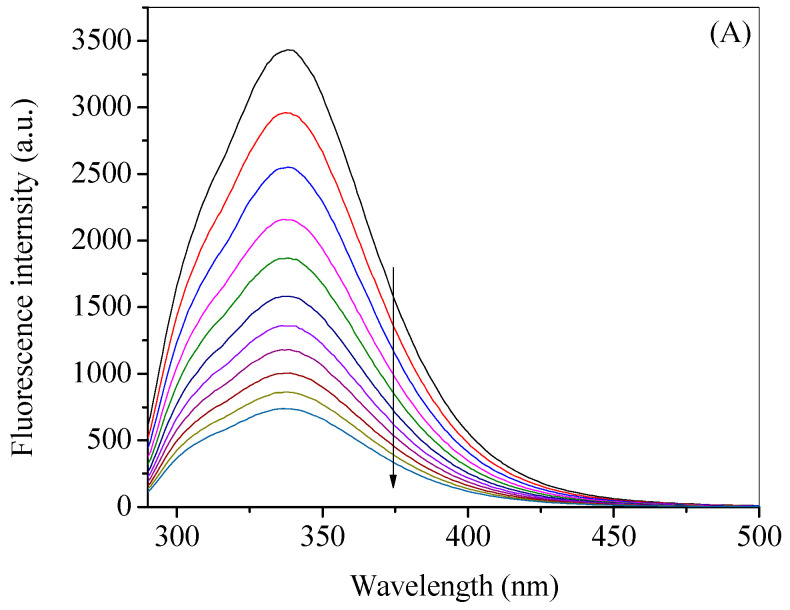

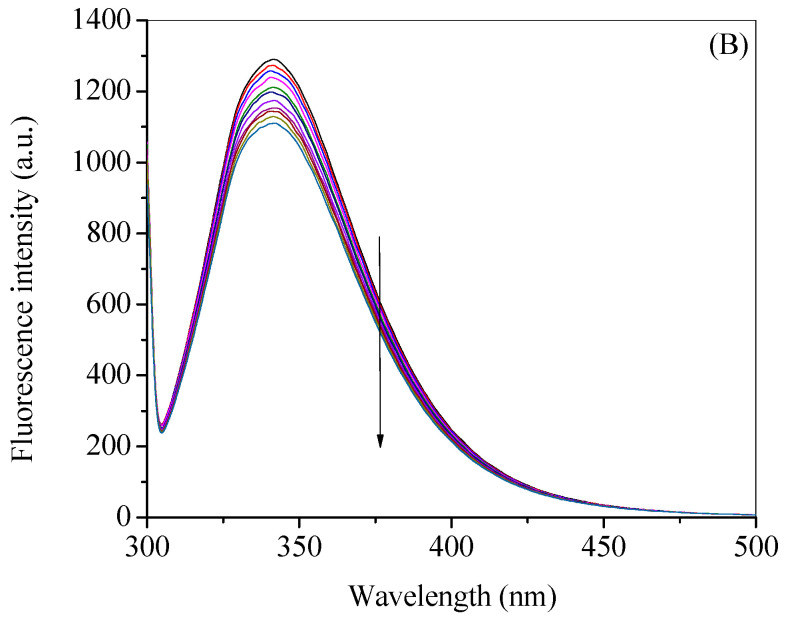
Observed fluorescence emission spectra of HSA (3 µM) at the excitation wavelengths of (**A**) 280 nm and (**B**) 295 nm in the presence of various concentrations of gemcitabine (0, 20, 40, 60, 80, 100, 120, 140, 160, 180 and 200 µM) at 20 °C in 20 mM tris buffer with pH 7.4.

**Figure 3:**
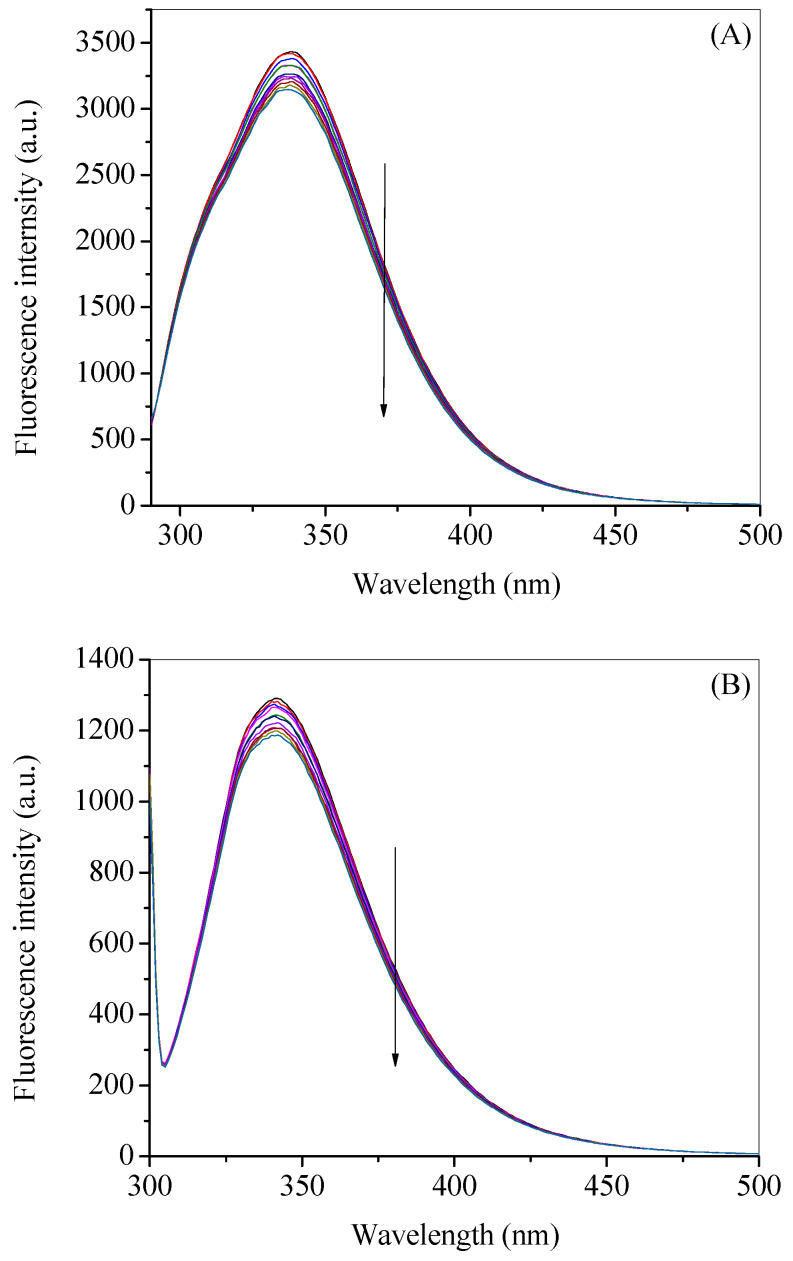
Corrected fluorescence emission spectra of HSA (3 µM) at the excitation wavelengths of (**A**) 280 nm and (**B**) 295 nm in the presence of various concentrations of gemcitabine (0, 20, 40, 60, 80, 100, 120, 140, 160, 180 and 200 µM) at 20 °C in 20 mM tris buffer with pH 7.4.

Fluorescence spectroscopy is an important technique to understand the various types of proteins interactions due to their intrinsic fluorescence properties, if the fluorescent amino acids like tryptophan and tyrosine are present in its backbone [43,44,45,46]. When both of these amino acids are present, tryptophan is the main contributor of the fluorescence emission while tyrosine has small or negligible contribution [47]. The common property of both tryptophan and tyrosine is that both gives off emissions at 340 nm and 315 nm, respectively, when excited at 280 nm, whereas tryptophan emission can be separated by exciting the protein at 295 nm [47]. The fluorescence emission spectra of HSA at 20 °C in the absence and presence of gemcitabine at respective excitation wavelength of 280 nm and 295 nm are given in Figure 2A,B. It can be seen from these figures that when gemcitabine is gradually added to the protein solution, the fluorescence decrement or quenching happens due to the interaction between these two. A visual examination of these figures shows that quenching is very large and even multifold when the excitation wavelength is 280 nm in comparison to the quenching observed in case of 295 nm excitation. However, it can be seen from the Figure 1A that gemcitabine has significant absorption at the excitation wavelengths, particularly at 280 nm; thus, the fluorescence data in presence of gemcitabine need to be corrected for the inner filter effect. Thus, we have corrected the observed fluorescence spectra (Figure 3A,B) using Equation (S1) given in Supplementary Material for both excitation wavelengths. After correction, it is noticeable that the extent of quenching is almost same in both cases and that there is low affinity between HSA and gemcitabine. The observed and corrected fluorescence spectra at both excitation wavelengths and at various temperatures (30 °C, 40 °C and 50 °C) are given in Figures S1–S6 of Supplementary Material. The studies at various temperatures have been carried out to understand the quenching mechanism and to evaluate the thermodynamic parameters.

### 2.3. Evaluation of Quenching and Thermodynamic Parameters

The Stern–Volmer equation was utilized to calculate the Stern–Volmer quenching constant (*K_SV_*):

(1) $$\frac{F_{0}}{F}=1+K_{SV}\left[Q\right]=1+K_{q}\tau_{0}\left[Q\right]$$

(2) $$K_{q}=\frac{K_{SV}}{\tau_{0}}$$

where *F*_0_ and *F* are the fluorescence intensities of HSA in the absence and presence of gemcitabine; [*Q*] is the concentration of gemcitabine, and *K_SV_*, *K_q_* and *τ*_0_ are the Stern–Volmer quenching constant, the bimolecular quenching constant and the life-time of the fluorophore in the absence of the quencher (in this case 5.7 × 10^–9^ s^–1^ according to [26]), respectively.

**Figure 4:**
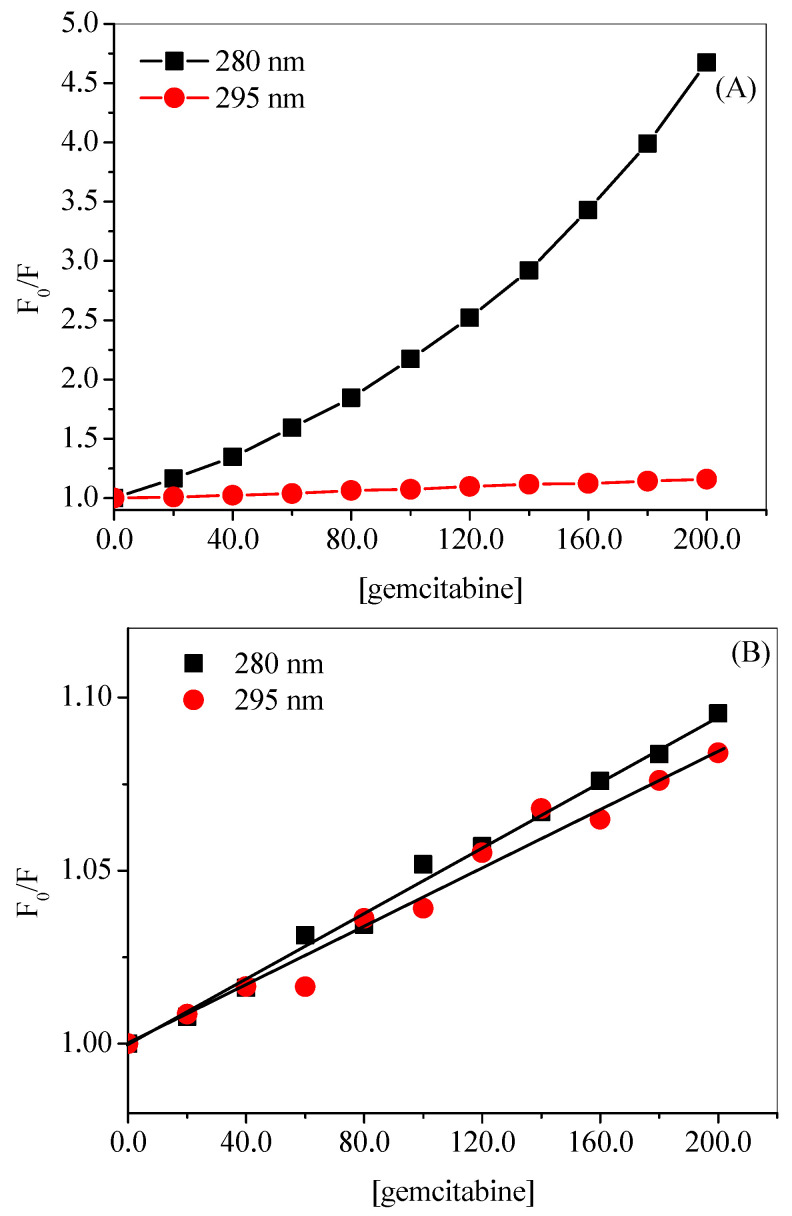
Plots of *F*_0_/*F* vs. [gemcitabine] for observed (**A**) and corrected data (**B**) for HSA–gemcitabine interaction at 20 °C in 20 mM tris buffer with pH 7.4. [HSA] = 3 µM.

For the sake of comparison of the observed and corrected results, we have calculated the *K_SV_* for both excitation wavelengths using the plots given in Figure 4A,B for observed and corrected data, respectively. The corresponding values of *K_SV_* at 280 nm excitation for observed and corrected data are found to be 152.7 × 10^2^ M^–1^ and 4.7 × 10^2^ M^–1^, whereas at 295 nm excitation the respective values are 7.9 × 10^2^ M^–1^ and 4.3 × 10^2^ M^–1^. There is a large diminution in the values of *K_SV_* after inner filter effect correction, mainly, at 280 nm excitation; although an around two-fold decrease is also observed in the case of 295 nm excitation. This could be explained on the basis of the values of the absorbance of gemcitabine at these two wavelengths, because, at 280 nm, the absorbance is much higher, which resulted in the larger inner filter effect, whereas small absorbance at 295 nm causes only small change. These observations support the fact that higher the absorbance of the ligand at the excitation and/or emission wavelengths higher will be the inner filter effect which may affect the actual results. Since there is not much difference between the quenching constants obtained from the corrected data at two excitation wavelengths (280 nm and 295 nm), we have selected 295 nm excitation for further studies.

**Figure 5:**
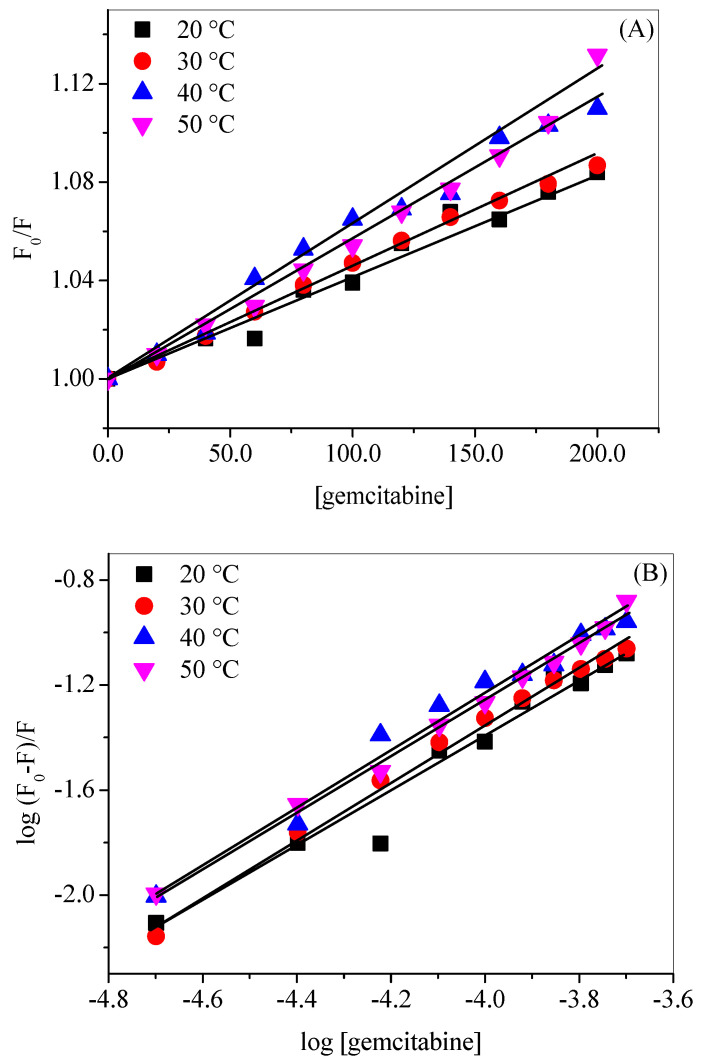
(**A**) Plots of *F*_0_/*F* vs. [gemcitabine] at and (**B**) plots of log (*F*_0_ − *F*)/F vs. log [gemcitabine] for HSA–gemcitabine interaction in 20 mM tris buffer with pH 7.4 at various temperatures. [HSA] = 3 µM.

The fluorescence quenching of a fluorophore by a quencher might be the result of several interactions, such as dark non-fluorescent complex formations called static quenching or collisional encounters between the former and latter, termed dynamic quenching [43,48]. A temperature change can differentiate between these two types of quenchings because static quenching increases with decreasing temperature while the reverse phenomenon happens in case of dynamic quenching [43]. The values of *K_q_* also play an important role in understanding the type of quenching involved in the binding. If the value of *K_q_* is near 1 × 10^10^ M^–1^ s^–1^ (the diffusion-controlled limit), the quenching is expected to be dynamic, whereas static quenching is generally associated with very high values of *K_q_* [47]. The Stern–Volmer plots (*F*_0_/*F* vs. [gemcitabine]) at various temperatures are given in Figure 5A, and the values of the *K_SV_* and *K_q_* are given in Table 1. The values of *K_SV_* increased slightly with increases in the temperature, and *K_q_* values, although somewhat higher than the diffusion-controlled limit, are of the same order of magnitude, as expected in cases of dynamic quenching, and are very low as previously discovered in the case of interactions taking place via static quenching. Thus, it can be said that HSA–gemcitabine binding takes place through dynamic quenching mechanism [36,49].

The binding constant (*K_b_*) and number of binding sites (*n*) can be calculated using the following equation [50]:

(3) $$log\frac{F_{0}-F}{F}=logK_{b}+nlog\left[Q\right]$$

The plots of log (*F*_0_ − *F*)/*F* versus log [*Q*], which have been used to calculate the *K_b_* and *n*, are given in Figure 5B, and the values of these parameters are given in Table 2. The magnitude of binding constants shows that there is a 1:1 binding with low affinity between HSA and gemcitabine. However, the values of n suggested that the binding is cooperative in nature [51,52].

**Figure 6:**
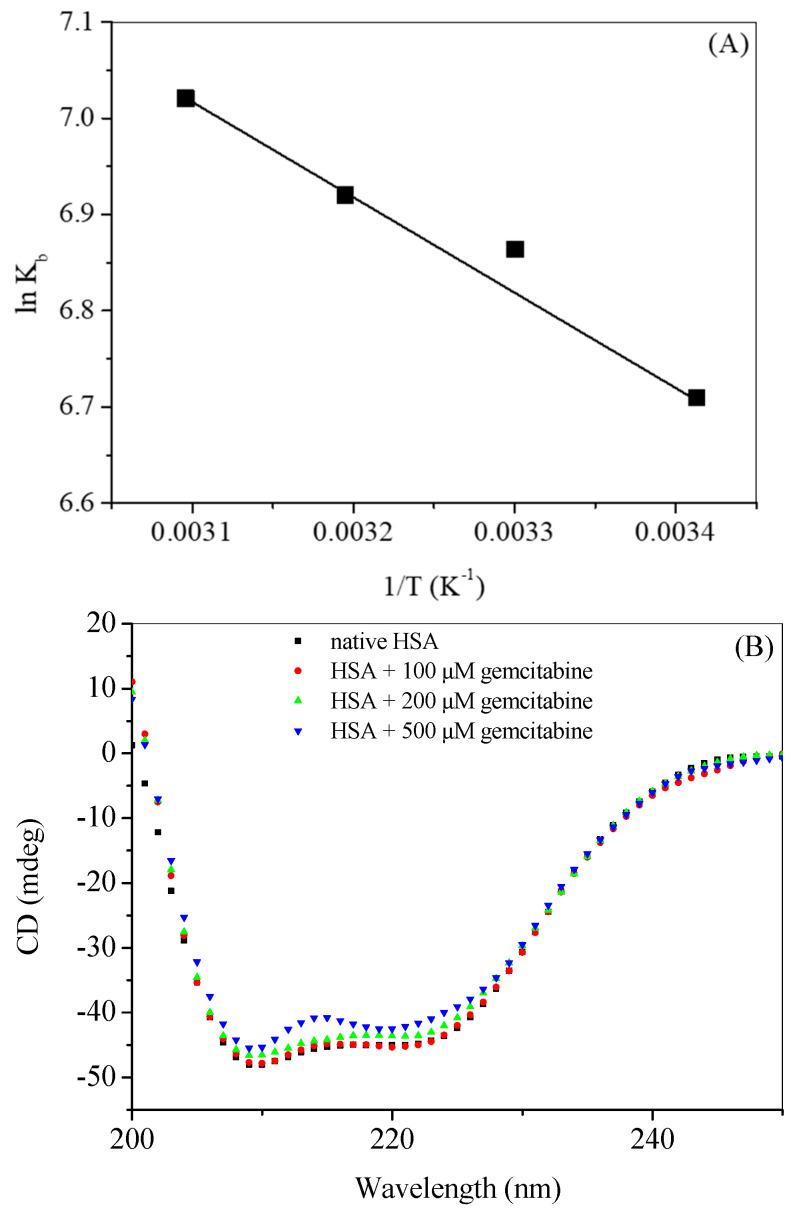
(**A**) van’t Hoff plot of HSA–gemcitabine interaction (**B**) Far-UV CD spectra of HSA in the absence and presence of gemcitabine in 20 mM tris buffer with pH 7.4 at various temperatures. [HSA] = 3 µM.

In a process of interaction between a biomolecule and small ligand, various binding forces, such as hydrogen bonds and electrostatic, hydrophobic and van der Waals forces are involved. Thermodynamic parameters like free energy change (Δ*G*), enthalpy change (Δ*H*) and entropy change (Δ*S*) can suggest the spontaneity of the binding as well as give an idea about the major forces involved in the binding. Van’t Hoff equations (Equations (S2) and (S3) given in supplementary material) can be utilized to calculate the thermodynamic parameters (Table 2) using the plot of ln *K_b_* vs. 1/*T*, which is given in Figure 6A. The dominance of hydrophobic interaction is associated with positive values of both Δ*H* and entropy change Δ*S*, while their negative values correspond to the hydrogen bonding and van der Waals forces; electrostatic interactions, on the other hand, play an important role when the values of Δ*H* is very low or zero. The negative values of Δ*G* (Table 1) suggest that the binding is spontaneous, which is favored by the temperature change. The calculated values of Δ*H* and Δ*S* show that the major binding forces associated with HSA–gemcitabine binding are hydrophobic forces [53].

### 2.4. Secondary Structural Analysis Using Far-UV CD Spectroscopy

The secondary structural changes in a protein can be estimated by CD spectrophotometry. Proteins have several secondary structural components for example, α-helix, anti-parallel β-sheet, β-turn, random coil, etc., and information on these types of structures can be obtained by the use of far-UV CD typically in the wavelength range of 200 nm to 250 nm [54,55]. HSA is an α-helical protein with about 67% of α-helical contents, which can be characterized by two negative peaks at 222 nm and 208 nm [56,57]. The far-UV CD spectra of native HSA and HSA–gemcitabine complex are given in Figure 6B. Low concentrations (up to 10 µM) of gemcitabine did not significantly affect the secondary structure of HSA; however, a small decrease in the α-helicity happens when the concentration of gemcitabine is increased up to 200 µM, and a further drop in the helicity can be observed in presence of 500 µM of the drug. Thus, it can be deduced here that, in the presence of lower concentrations (up to 100 µM) of gemcitabine, the secondary structure of HSA remained intact, while higher concentrations of the former give rise to the partial unfolding of the latter.

### 2.5. Competitive Binding Site Experiments and Effect of Ibuprofen on the Binding

**Figure 7:**
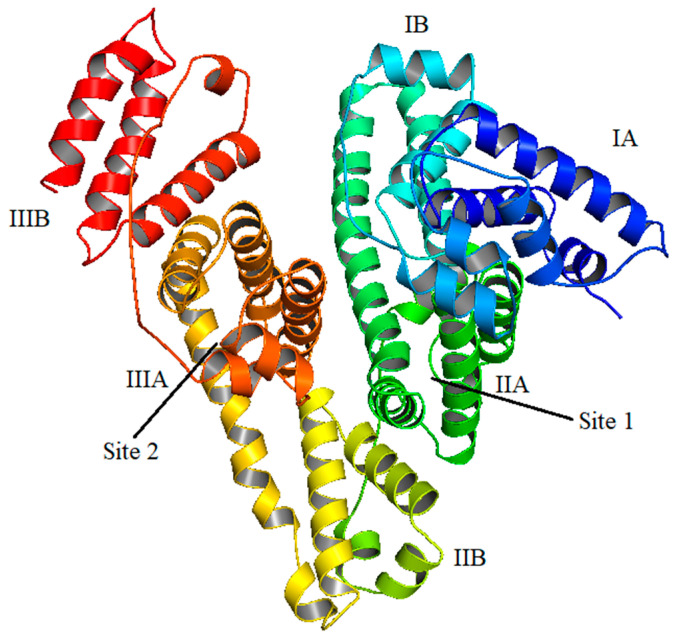
Schematic representation of HSA showing various sub-domains and the two principal binding sites.

**Figure 8:**
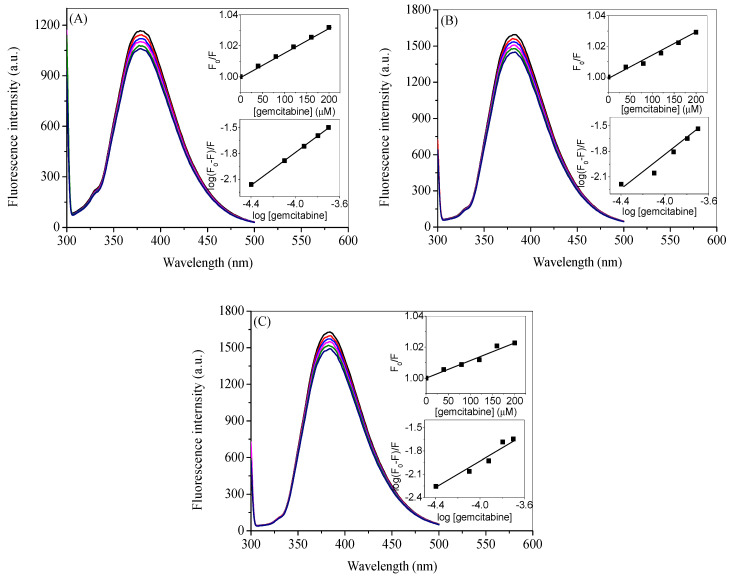
Observed fluorescence emission spectra of HSA complexed with warfarin at the excitation wavelength of 295 nm in the presence of various concentrations of gemcitabine (0, 20, 40, 60, 80, 100, 120, 140, 160, 180 and 200 µM) at 20 °C in 20 mM tris buffer with pH 7.4. [HSA] = 3 µM, [warfarin] = 3 µM (**A**), 10 µM (**B**) and 20 µM (**C**). The upper insets in each figure show the plots of *F*_0_/*F* vs. [gemcitabine], and lower insets show the plots of log (*F*_0_ − *F*)/*F* vs. log [gemcitabine] obtained from the data after the inner filter effect correction of the respective figure.

**Figure 9:**
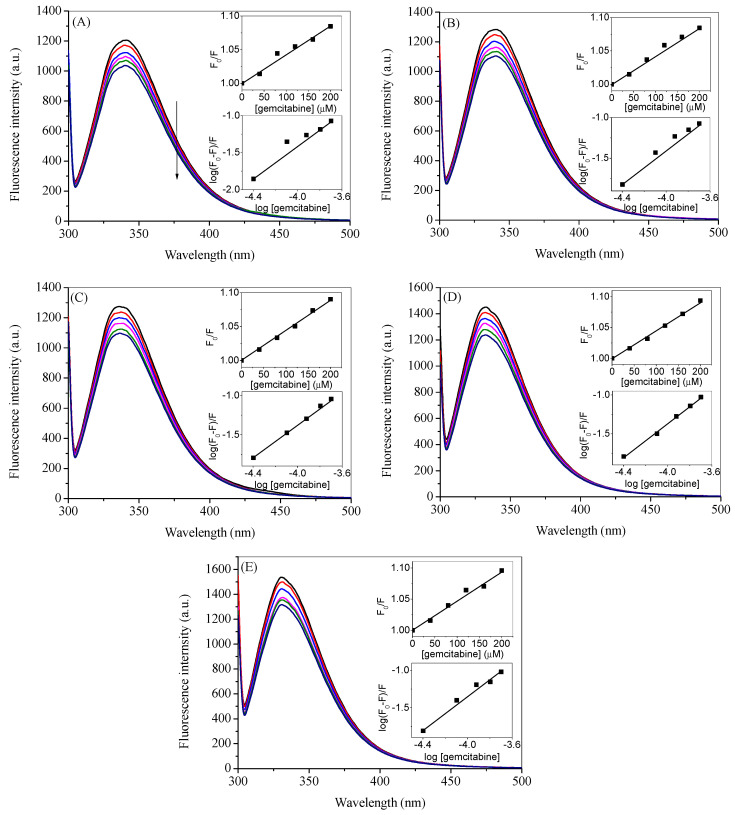
Observed fluorescence emission spectra of HSA complexed with ibuprofen at the excitation wavelength of 295 nm in the presence of various concentrations of gemcitabine (0, 20, 40, 60, 80, 100, 120, 140, 160, 180 and 200 µM) at 20 °C in 20 mM tris buffer with pH 7.4. [HSA] = 3 µM, [ibuprofen] = 3 µM (**A**), 10 µM (**B**), 20 µM (**C**,**D**) 100 µM and (**E**) 200 µM. The upper insets in each figure show the plots of *F*_0_/*F* vs. [gemcitabine], and lower insets show the plots of log (*F*_0_ − *F*)/*F* vs. log [gemcitabine] obtained from the data after the inner filter effect correction of the respective figure.

HSA is a globular protein with around 585 amino acids with molecular mass of 66.5 kDa. Structurally, it is divided into three domains (I, II and III), each of which are further subdivided into two subdomains (A and B) as shown in Figure 7 [58]. According to Sudlow et al., there are two principal binding sites in HSA for the drugs that are located in subdomain IIA and IIIA and are characterized as drug site 1 and drug site 2. It was reported by Ghuman et al. that warfarin binds specifically at drug site 1, whereas ibuprofen binds at drug site 2, although the latter can also bind at another site, which is termed its secondary site [59]. Keeping in mind the established drug-binding sites, competitive binding site experiments were carried out using warfarin as a marker for drug site 1 and ibuprofen as a marker for drug site 2. Moreover, since ibuprofen does not influence the binding significantly at a lower concentration range (vide infra) and considering the possibility of its coexistance with gemcitabine in the prescription (as described in introduction section), we have seen the effect of additional higher concentrations of ibuprofen on the binding of gemcitabine with HSA. The fluorescence quenching of HSA with gemcitabine was studied in the presence of these site markers and the respective spectra are given in Figure 8 and Figure 9. In these figures, we have only displayed the observed spectra, though the data were corrected for the inner filter effect before the analysis of the *K_SV_* and *K_b_* (insets of Figure 8 and Figure 9). We have used several concentrations of both site markers to understand the effect of their concentrations on the interactions between HSA and gemcitabine. The quenching and binding constants greatly decrease in the presence of small concentrations of warfarin (Table 3), which is due to the competition and/or the steric hindrence that happened between gemcitabine and the former for the same site, which resulted in decreasing the binding affinity of latter. There is almost no effect of small, as well as higher, concentrations of ibuprofen on the binding of HSA and gemcitabine (Table 4).

From these outcomes, it can be understood that the binding site of gemcitabine inside HSA is near the binding site of warfarin, i.e., drug site 1. Interestingly we observe that ibuprofen does not influence HSA–gemcitabine binding.

### 2.6. Detailed Molecular Docking Studies of HSA Interaction with Gemcitabine in the Absence and Presence of Site Markers

**Figure 10:**
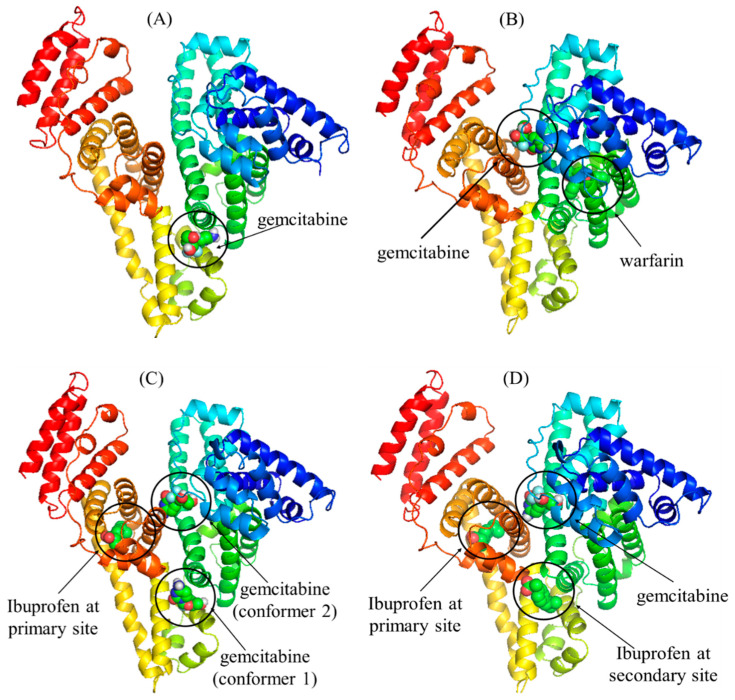
In silico molecular docking poses of gemcitabine inside HSA under various conditions: (**A**) unliganded HSA, (**B**) HSA–warfarin complex, (**C**) HSA–ibuprofen complex at the primary site and (**D**) HSA–ibuprofen complex at the primary, as well as secondary, sites.

**Figure 11:**
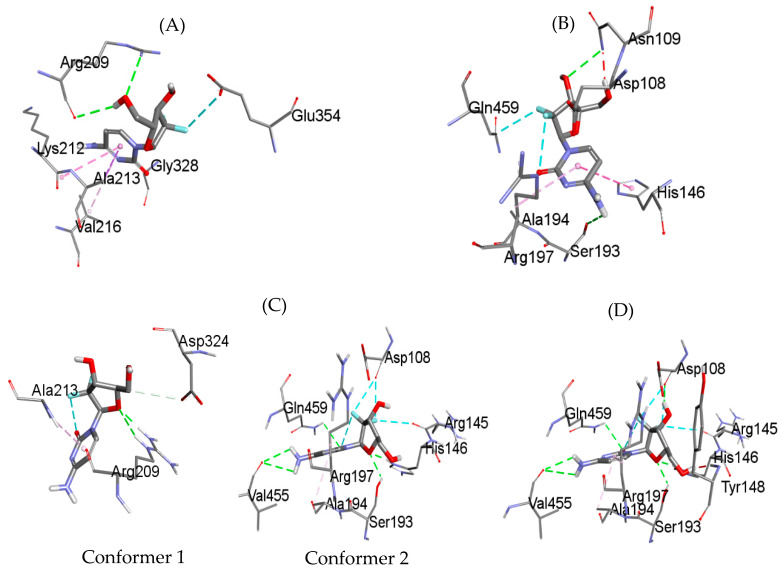
Binding pocket analyses of gemcitabine inside HSA under various conditions: (**A**) unliganded HSA, (**B**) HSA–warfarin complex, (**C**) HSA–ibuprofen complex at primary site showing two conformers of the same energies and (**D**) HSA–ibuprofen complex at primary as well as secondary sites.

For a better understanding of the HSA–gemcitabine interaction, molecular docking was also carried out to locate the binding site of gemcitabine inside free HSA, HSA complexed with warfarin and ibuprofen (both primary and secondary sites). The docked structures of gemcitabine with free HSA and HSA pre-complexed with warfarin and ibuprofen, obtained using Autodock vina, are given in Figure 10. In the unliganded HSA, gemcitabine preferred to bind at the interface of subdomain IIA and IIB (Figure 9A), which is in close proximity to the warfarin binding site and also near to the secondary site for ibuprofen [59]. In warfarin-bounded HSA (at site 1), the preferred binding site of gemcitabine is near the cleft at the interfacial region of domain IB and domain IIIA (Figure 10B) due to the possible steric hindrance and/or competition with the warfarin complexed in the near proximity. As ibuprofen is known to have two binding sites inside HSA, we have also seen the preferred binding site of gemcitabine when only primary as well as when both sites are occupied by ibuprofen. When one molecule of ibuprofen is complexed with HSA inside subdomain IIIA, the docking results of gemcitabine show two conformers with the same docking score (Figure 10C). The location of gemcitabine in the first conformer is the same as that observed in free HSA, while in the second conformer, the binding site is located at the cleft (the same site observed in the case of warfarin-complexed HSA). In the case of occupancy of both primary as well as secondary sites by ibuprofen, the gemcitabine binding site is also the same, as observed with warfarin-HSA (Figure 10D). The interesting thing to note here is the docking score of Autodock vina, which is −7.0 kcal/mol and −6.6 kcal/mol for the free HSA and warfarin complexed HSA. In the case of the docking of gemcitabine with HSA complexed with ibuprofen at only the primary site, the docking score remains the same as in free HSA for both conformers described above, whereas it increases to −7.2 kcal/mol for the binding of gemcitabine with the HSA bonded with two molecules of ibuprofen. These results are in good agreement with the results obtained experimentally, which shows that ibuprofen did not affect the binding of gemcitabine with HSA. The binding pockets of gemcitabine with HSA (free as well as complexed with site markers) are given in Figure 11, and the amino acids involved in the binding, as well as the type of interaction, are displayed in Table 5.

### 2.7. Density Functional Theory (DFT) Studies

Though the DFT studies of gemcitabine alone using Gaussian package have been reported earlier [60], we have investigated the DFT studies of various conformers described in the molecular docking section using the ORCA package. The frontier molecular orbitals (FMOs) (highest occupied molecular orbital (HOMO) and lowest unoccupied molecular orbital (LUMO)) of free and complexed gemcitabine are displayed in Table 6. The energy gap (ΔE) between the HOMOs and LUMOs gives an idea about the stability of a molecule. It can be seen from the values of ΔE given in Table 6 that there is not much difference in the values corresponding to various forms of gemcitabine, although the spatial arrangements of bonds as well as atoms within the molecule is different in each form, which is due to the flexibility of gemcitabine, due to which it fits inside the binding pocket of HSA [26]. Generally, a molecule with a small frontier orbital gap is more polarizable and has high chemical reactivity and low kinetic stability [61], whereas a larger gap leads to a greater molecular stability for further reactions [62,63,64]. The slight difference in ΔE observed in the present case might be due to the weaker interaction between HSA and gemcitabine because, in stronger interactions, a considerable increase in the ΔE was reported [21]. However, an in-depth comparison of the ΔE of gemcitabine under various conditions of binding shows that gemcitabine was least stable when bonded with the HSA–warfarin complex; in intermediate stability range in free form, it bonded with HSA that was free from any other ligand and bonded with the HSA–ibuprofen complex at the primary site; nevertheless, the stability of gemcitabine slightly increased further when it bonded with HSA containing ibuprofen at the primary as well as secondary sites of HSA.

## 3. Materials and Methods

HSA and gemcitabine were the products of Sigma USA and were used as received. The studies were carried out in 20 mM tris buffer with pH 7.4 at 20 °C unless stated otherwise. A protein concentration of 3.0 µM was used in most of the experiments. UV-visible spectra were collected using a Perkin-Volmer Lambda-45 Spectrophotometer. Fluorescence measurements were performed using a Hitachi F-7000 spectrofluorometer, which was connected with a programmer temperature controller unit. The fluorescence emission spectra were collected by exciting the protein solution at 280 nm and 295 nm using the excitation and emission slit width of 5 nm and with PMT voltage of 500 V. CD spectra of HSA was screened using a Jasco J-815 spectropolarimeter in the far-UV range (200 to 250 nm). Molecular docking simulations were performed using the Autodock vina program [65]. The structural coordinates of HSA and gemcitabine (PubChem CID 60750) were obtained from Pubmed and PubChem databases, respectively, and prepared using discovery studio visualizer program. In the case of molecular docking of free HSA and gemcitabine, ligand-free HSA structure (4K2C) was selected, and, for other dockings, i.e., warfarin-bounded (2BXD) and ibuprofen-bounded (2BXG), structures were chosen. The rest of the details of the experimental methodology are given in the supplementary material. The geometry of gemcitabine was optimized at DFT/B3LYP/6-31 using the ORCA program [66], and the analysis and visualization were performed using the Avogadro software [67].

## 4. Conclusions

Gemcitabine is a popular anticancer drug that is prescribed to patients for the treatment of various types of cancers. The interaction of gemcitabine with plasma proteins is prospective during its administration; therefore, its interaction with the most-abundant plasma protein was studied in this paper. The possibility of the copresence of the well-known painkiller, ibuprofen, along with gemcitabine, was also considered, and the effect of the former was also seen on the binding. The binding of gemcitabine with HSA was found to be weak, which increased with increases in the temperature due to the dynamic nature of the quenching process. The secondary structure of HSA did not change in the presence of a low concentration of gemcitabine; although, partial unfolding was observed when the drug was present in larger amounts. The preferred binding site of gemcitabine was near drug binding site 1, located at the interface of subdomain IIA and IIB. There was apparently no effect of ibuprofen on the binding of gemcitabine with HSA, even in the presence of higher concentrations of the former. Thus, it could be concluded here that ibuprofen does not influence the binding of gemcitabine.

## Figures and Tables

**Table 1:** Quenching parameters for the interaction of HSA with gemcitabine obtained from the corrected data at various temperatures.

Temperature (°C)	10^2^ *K_sv_* (mol^−1^)	10^10^ *k_q_* (mol^−1^∙s^−1^)	*R* ^2^
	λex = 280 nm		
20	4.7	8.0	0.9945
	λex = 295 nm		
20	4.3	7.2	0.9768
30	4.5	7.6	0.9955
40	5.8	9.8	0.9827
50	5.9	9.9	0.9819

**Table 2:** Binding and thermodynamic parameters for the interaction of HSA with gemcitabine at various temperatures.

Temperature	Binding Parameters			Thermodynamic Parameters		
	*n*	10^2^*K_b_* (mol^−1^)	*R* ^2^	∆*G* (kJ mol^−1^)	∆*H* (kJ mol^−1^)	∆*S* (J mol^–1^ K^−1^)
20	1.1	8.2	0.963	−16.4	7.8	82.6
30	1.1	9.6	0.9924	−17.2		
40	1.1	10.1	0.9784	−18.1		
50	1.1	11.2	0.9952	−18.9		

**Table 3:** Stern–Volmer quenching constants and the binding constants of HSA–gemcitabine interaction in the presence of various amounts of warfarin.

[warfarin] (µM)	*K_sv_* (M^−1^)	*K_b_* (M^−1^)
3.0	1.6 × 10^2^	1.0 × 10^2^
10.0	1.4 × 10^2^	0.9 × 10^2^
20.0	1.1 × 10^2^	0.5 × 10^2^

**Table 4:** Stern–Volmer quenching constants and the binding constants of HSA–gemcitabine interaction in the presence of various amounts of ibuprofen.

[ibuprofen] (µM)	*K_sv_* (M^−1^)	*K_b_* (M^−1^)
3.0	4.3 × 10^2^	7.5 × 10^2^
10.0	4.4 × 10^2^	7.3 × 10^2^
20.0	4.4 × 10^2^	7.2 × 10^2^
100.0	4.3 × 10^2^	7.0 × 10^2^
200.0	4.2 × 10^2^	7.1 × 10^2^

**Table 5:** Non-covalent interactions of gemcitabine with free and bounded HSA obtained through molecular docking.

	Amino Acid	Type of Interaction
free HSA	ARG209	hydrogen bonding
	GLY328	
	ALA213	hydrophobic interaction
	LYS212	
	VAL216	
	GLU354	halogen acceptor
HSA bounded with warfarin	SER193	hydrogen bonding
	ASN109	
	ASP108	
	ALA194	
	GLN459	
	HIS146	halogen acceptor
	ARG197	hydrophobic interaction
	SER193	
HSA bounded with ibuprofen at primary site	Conformer 1	
	ARG209	hydrogen bonding
	ASP324	
	ALA213	hydrophobic interaction
	Conformer 2	
	VAL455	hydrogen bonding
	HIS146	
	SER193	
	GLN459	
	ASP108	halogen acceptor
	ARG145	
	ALA194	hydrophobic interaction
	ARG197	
HSA bounded with ibuprofen at primary and secondary sites	VAL455	hydrogen bonding
	ASP108	
	HIS146	
	SER193	
	GLN459	
	ARG145	halogen acceptor
	ALA194	hydrophobic interaction
	ARG197	

**Table 6:** FMO diagrams and their energy gap for free and complexed gemcitabine obtained through geometry optimization using DFT.

	HOMO	LUMO	ΔE
Free gemcitabine	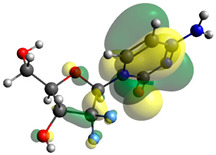	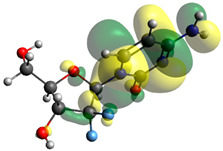	5.339
Gemcitabine docked with HSA	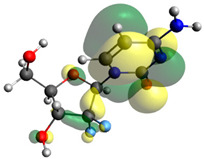	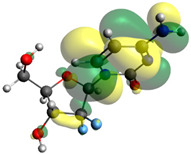	5.340
Gemcitabine docked with HSA–warfarin complex	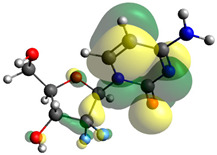	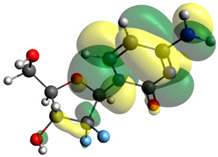	5.334
Gemcitabine docked with HSA complexed with ibuprofen at the primary site (conformer 1)	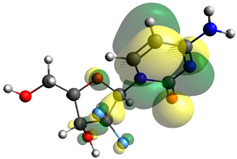	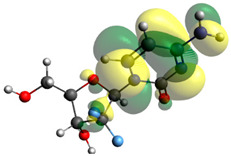	5.343
Gemcitabine docked with HSA complexed with ibuprofen at the primary site (conformer 2)	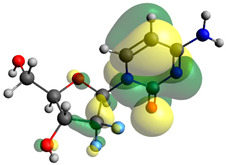	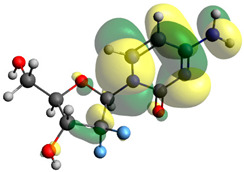	5.344
Gemcitabine docked with HSA complexed with ibuprofen at the primary as well as secondary sites	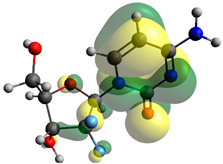	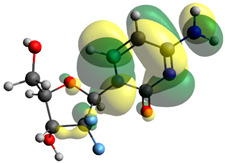	5.360

## Data Availability

Not applicable.

## Supplementary Materials

The following are available online, Figures S1–S3: Observed fluorescence spectra at various temperatures and Figures S4–S6: Corrected fluorescence spectra at various temperatures. References [47,65,68] are cited in the supplementary materials.

## Sample Availability

Samples of the compounds are not available from the authors.
