# Supplementary material for: Experimental and Computational Investigation on the Interaction of Anticancer Drug Gemcitabine with Human Plasma Protein: Effect of Copresence of Ibuprofen on the Binding
Source: Molecules. 2022 Mar 1;27(5):1635. doi: 10.3390/molecules27051635 (PMC8912049; doi:10.3390/molecules27051635)
Supplement: Supplementary file 1 [file molecules-27-01635-s001.zip › molecules-1554097-supplementary.pdf]

Supporting Information

UV–Visible spectra were recorded between the wavelength range of 200 to 400 nm on Perkin-Elmer Lambda 45 Spectrophotometer equipped with autosampler and water-bath with temperature controller. Quartz cuvettes of 1 cm path length were used for the measurements.

Fluorescence measurements were performed on Hitachi spectrofluorometer (Model F 7000) equipped with a PC and programmable temperature controller. Unless stated, the fluorescence spectra were collected at 20 °C with a cell of path length 1 cm. The excitation and emission slits were set at 5 nm. Intrinsic fluorescence was measured by exciting HSA at 295 nm.

The circular dichroism studies of HSA in presence of gemcitabine were carried out with JASCO J-815 spectropolarimeter equipped with a Peltier-type temperature controller. The instrument was calibrated with d-10-camphorsulfonic acid. All the CD spectra were collected in a cell of 2 mm path-length. The scan speed was 100 nm/min and response time of 1 s for all measurements. Each spectrum was the average of 3 scans.

The inner filter effect was corrected using the following equation [47]:

$$F_{corr} = F_{obs} \times 10^{(A_{exi} + A_{emi})/2} \quad \text{(S1)}$$

where, F_{corr} and F_{obs} are the corrected and observed fluorescence emission intensities, respectively, A_{exi} and A_{emi} are the absorbance at the excitation and emission wavelengths, respectively.

Equation used to calculate thermodynamic parameters:

van 't Hoff equation is given as:

$$\ln K_b = \frac{-\Delta H}{RT} + \frac{\Delta S}{R} \tag{S2}$$

$$\Delta G = \Delta H - T\Delta S \tag{S3}$$

where ΔH is enthalpy change, ΔS is entropy change and ΔG is free energy change. R is gas constant and T is temperature in K.

AutoDock Vina 1.1.2 (Molecular Graphics Lab, La Jolla, CA, USA) was used to see the possible binding mode of gemcitabine with HSA [65]. The 3D structures of ligand free HSA (PDB ID: 4K2C), warfarin bounded (2BXD) and ibuprofen bounded (2BXG) were obtained from the Protein Data Bank (PDB). The 3D structure of gemcitabine (pubchem CID 60750) was obtained from Pubchem database. The AutoDockTools 1.5.6 package (Molecular Graphics Lab, La Jolla, CA, USA) was used to generate the docking input files [68]. The grid was made in such a way that it covered the whole protein molecule and to get more accuracy, exhaustiveness of the rum was kept 1000 which allows gemcitabine molecule to move almost at every possible site at HSA. The least energy conformation was selected among 20 conformations obtained through docking.

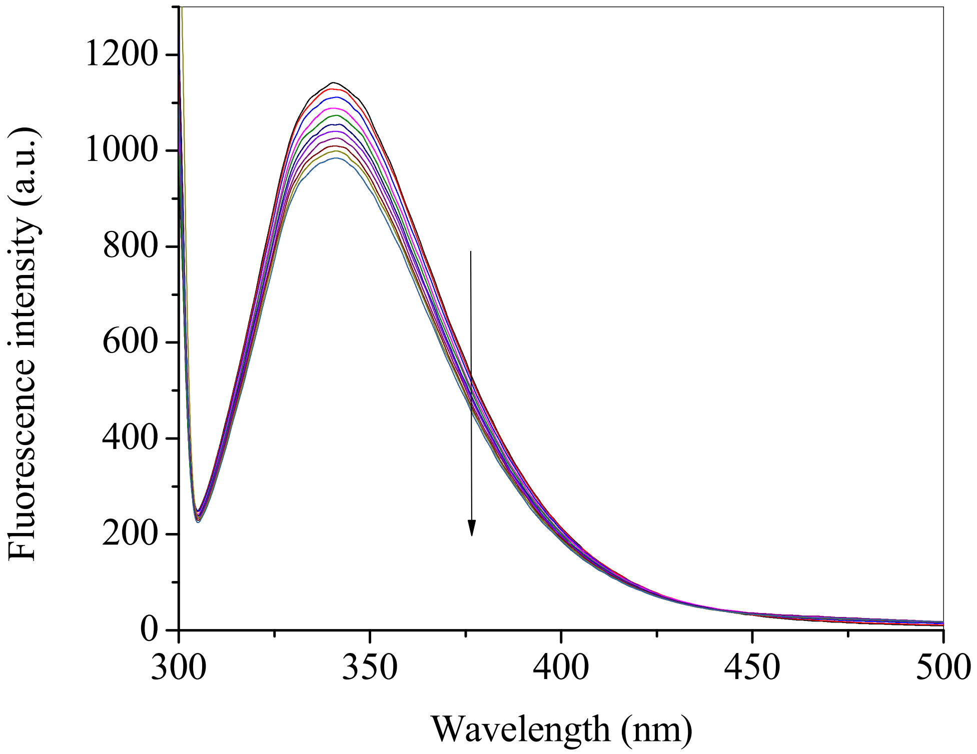

Figure S1. Observed fluorescence emission spectra of HSA at the excitation wavelength of 295 nm in presence of various concentrations of gemcitabine (0, 20, 40, 60, 80, 100, 120, 140, 160, 180 and 200 μM) at 30 °C.

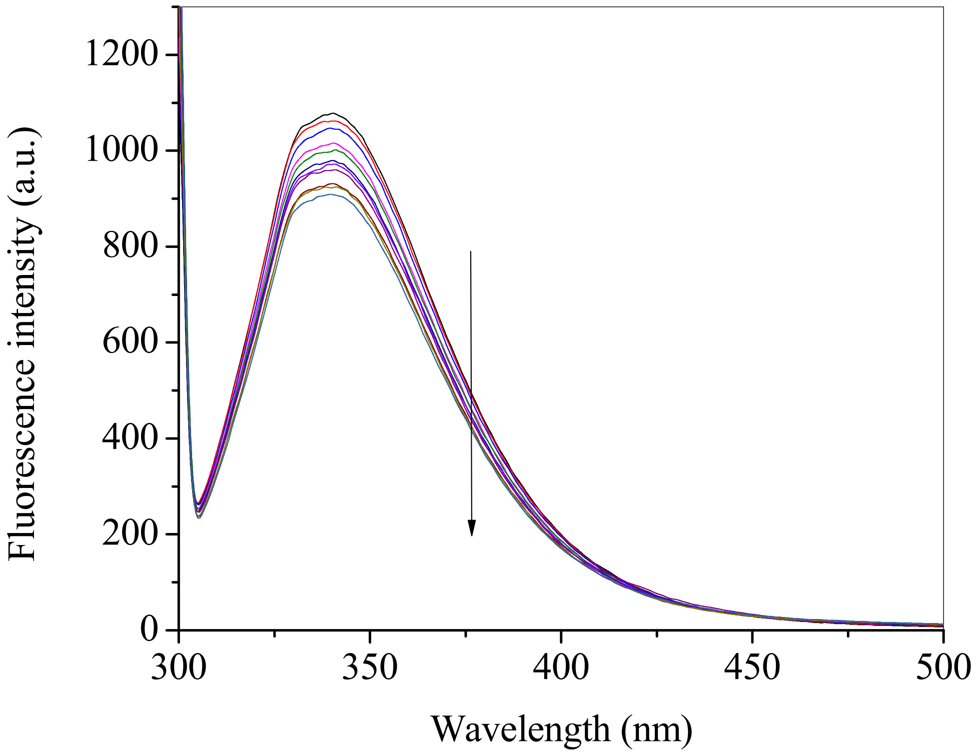

Figure S2. Observed fluorescence emission spectra of HSA at the excitation wavelength of 295 nm in presence of various concentrations of gemcitabine (0, 20, 40, 60, 80, 100, 120, 140, 160, 180 and 200 μM) at 30 °C.

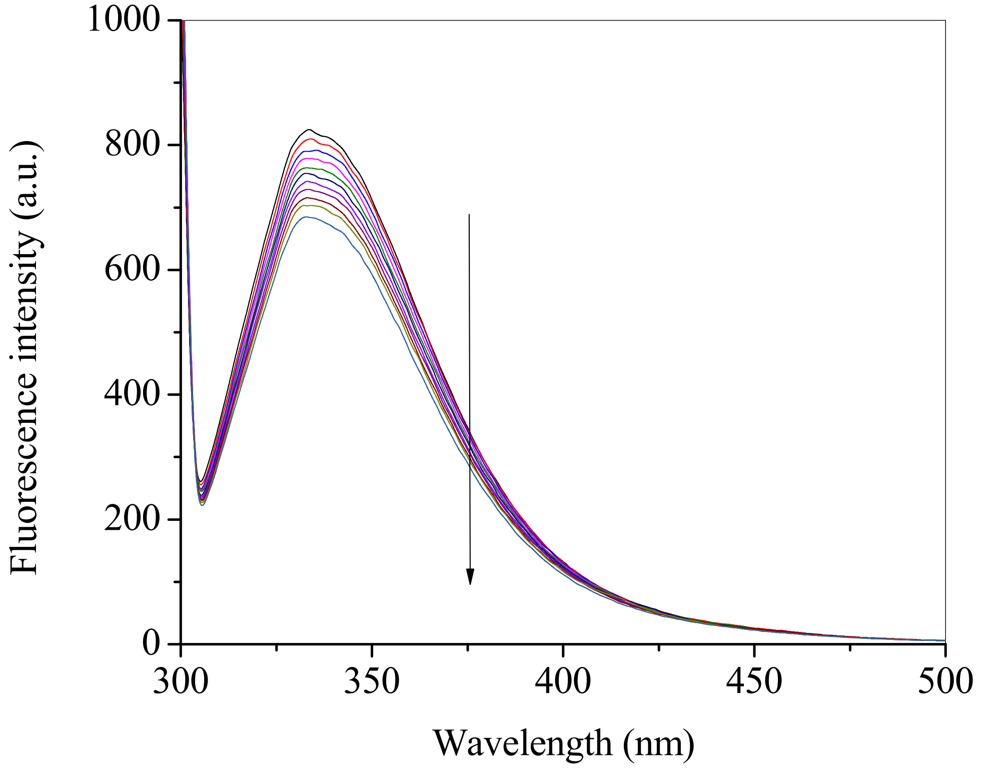

Figure S3. Observed fluorescence emission spectra of HSA at the excitation wavelength of 295 nm in presence of various concentrations of gemcitabine (0, 20, 40, 60, 80, 100, 120, 140, 160, 180 and 200 μM) at 50 °C.

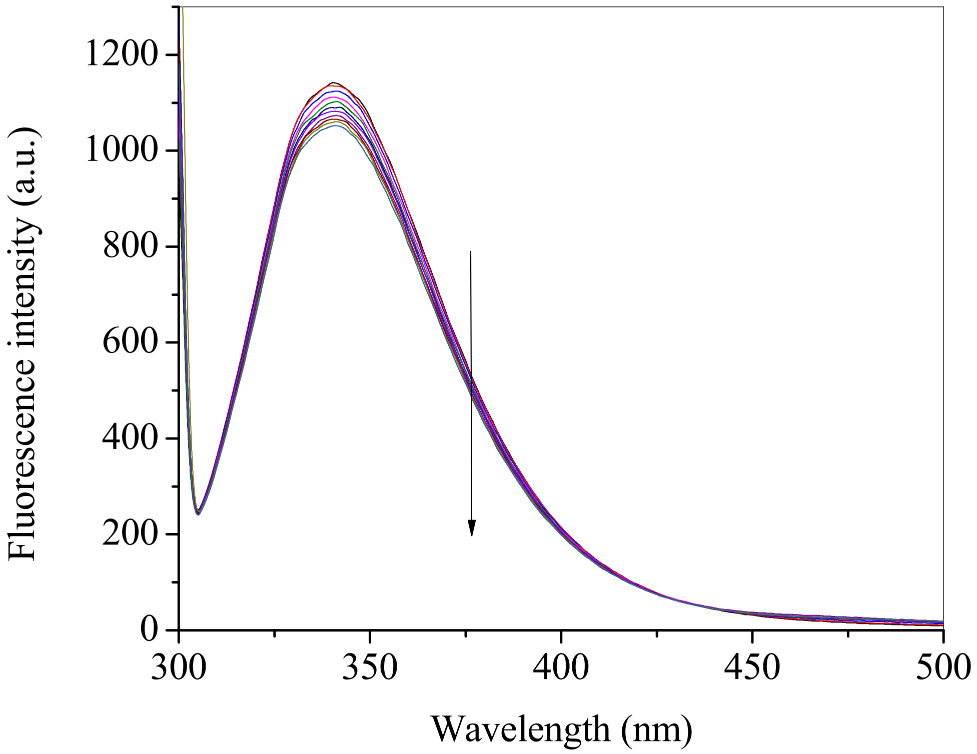

Figure S4. Corrected fluorescence emission spectra of HSA at the excitation wavelength of 295 nm in presence of various concentrations of gemcitabine (0, 20, 40, 60, 80, 100, 120, 140, 160, 180 and 200 μM) at 30 °C.

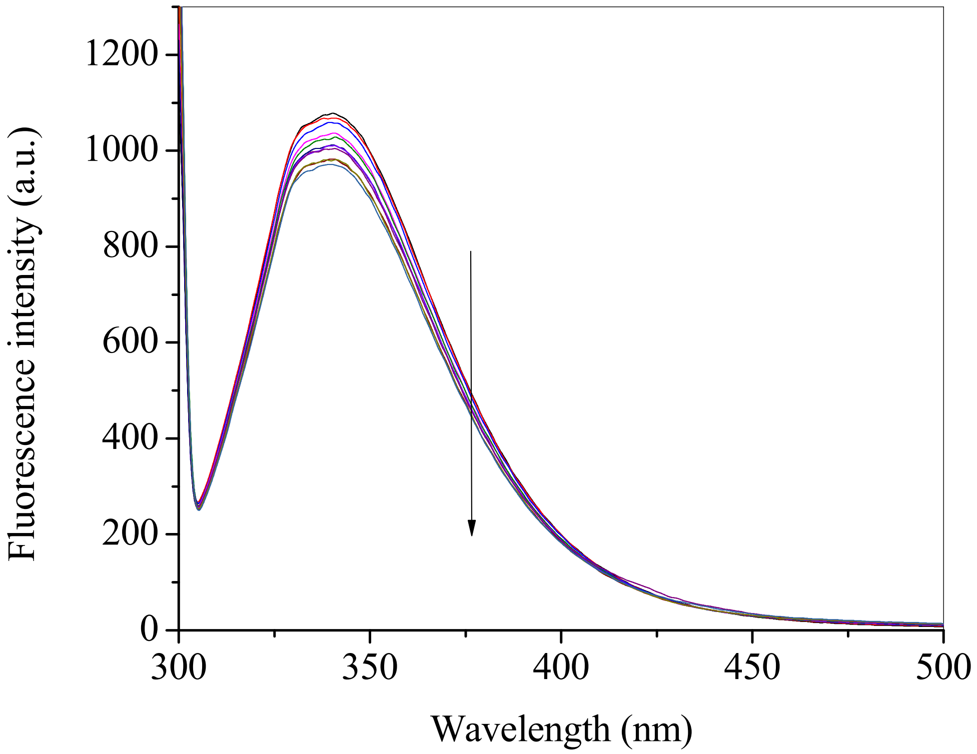

Figure S5. Corrected fluorescence emission spectra of HSA at the excitation wavelength of 295 nm in presence of various concentrations of gemcitabine (0, 20, 40, 60, 80, 100, 120, 140, 160, 180 and 200 μM) at 40 °C.

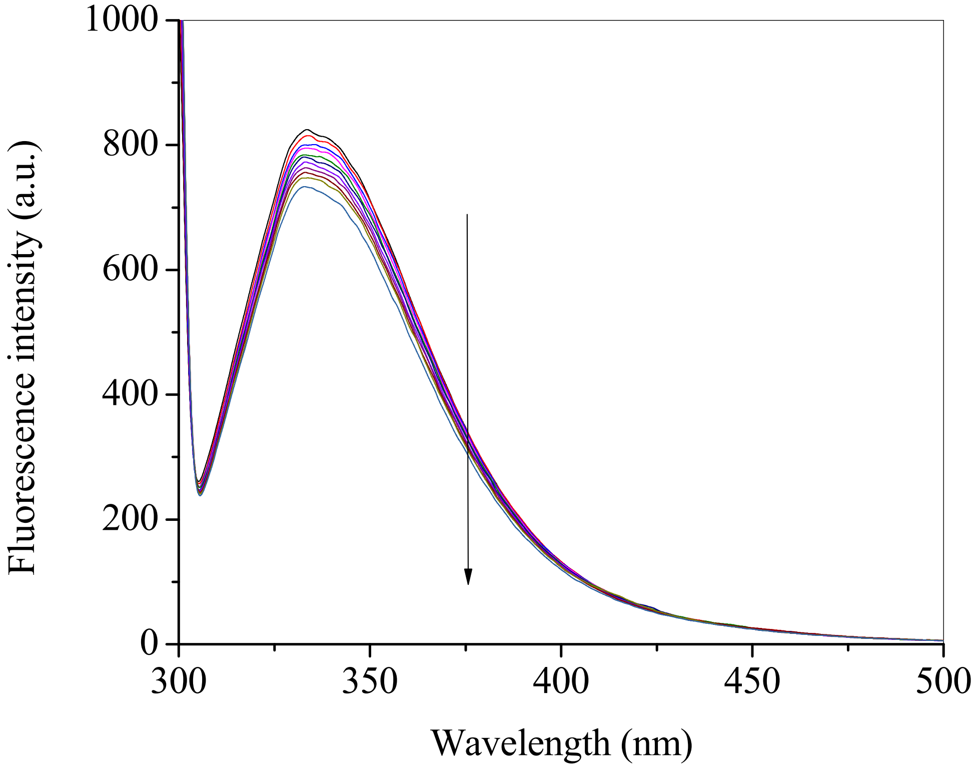

Figure S6. Corrected fluorescence emission spectra of HSA at the excitation wavelength of 295 nm in presence of various concentrations of gemcitabine (0, 20, 40, 60, 80, 100, 120, 140, 160, 180 and 200 μM) at 50 °C.
